# Critical Confluence: Gene Variants, Insecticide Exposure May Increase Childhood Brain Tumor Risk

**DOI:** 10.1289/ehp.118-a35a

**Published:** 2010-01

**Authors:** Julia R. Barrett

**Affiliations:** **Julia R. Barrett**, MS, ELS, a Madison, Wisconsin–based science writer and editor, has written for *EHP* since 1996. She is a member of the National Association of Science Writers and the Board of Editors in the Life Sciences

Epidemiologic data have suggested a link between pesticide exposures and childhood brain tumors. The link may be specific to insecticides such as organophosphorus and carbamate compounds, which are known to target the nervous system. Previously published work [*EHP* 113:909–913] investigated the role of individual genetic variation with a focus on paraoxonase (PON1), a key enzyme in the metabolism of organophosphorus insecticides commonly used in homes at the time but now banned for residential use. This work showed that children with brain tumors were more likely to carry a common single-nucelotide polymorphism (SNP) gene variant in the promoter region of the *PON1* gene (*PON1*_C-108T_) than other children, and that the association between this SNP and brain tumors was stronger in children with a history of home insecticide exposure. Research in an expanded study population now provides additional evidence that exposure to insecticides, paired with specific metabolism gene variants, may increase the risk of childhood brain tumors **[*EHP*** **118:144–149; Searles Nielsen et al.]**.

The research population included 201 children in California and Washington who had been diagnosed with a primary tumor of the brain, cranial nerves, or meninges between 1984 and 1991, as well as 285 children from the same geographic areas who served as controls. All children were aged 10 years or younger. Genetic information was extracted from archived dried blood spots used for routine screening tests when the children were born. In addition to *PON1*_C-108T_, the genetic analysis covered 7 other gene polymorphisms that might influence the children’s ability to metabolize insecticides. Interviews with the children’s mothers provided data on prenatal and childhood exposures to insecticides in the home.

Between the cases and controls, there was little difference in the prevalence of any of the polymorphisms. For cases, more of the mothers reported in-home insecticide use during pregnancy, but in-home treatment during childhood was more common among controls. Data analysis confirmed the original observation that children exposed to insecticides were more likely to have brain tumors if they also carried the *PON1*_C-108T_ SNP. Evidence of similar interactions also were observed with two other gene variants, *FMO1*_C-9536A_ and *BCHE*_A539T_, which also may affect the ability to detoxify organophosphorus and/or carbamate insecticides.

These findings suggest that children who are exposed to insecticides at a young age may have a greater risk of developing brain tumors if they carry these and possibly other polymorphisms. Larger studies are needed to confirm the findings, and environmental and biological measurements of specific pesticides, inclusion of more polymorphisms, and detailed information on exposure timing and dose would strengthen support for causal effects of insecticides and gene–environment interactions on the risk of childhood brain tumors.

